# Occurrence of Mycotoxins in Swine Feeding from Spain

**DOI:** 10.3390/toxins11060342

**Published:** 2019-06-15

**Authors:** Natalia Arroyo-Manzanares, Vicente Rodríguez-Estévez, Plácido Arenas-Fernández, Ana M. García-Campaña, Laura Gámiz-Gracia

**Affiliations:** 1 Department Analytical Chemistry, Faculty of Sciences, University of Granada, Campus Fuentenueva s/n, 18071 Granada, Spain; natalia.arroyo@um.es (N.A.-M.); placi_94_miercoles@hotmail.com (P.A.-F.); amgarcia@ugr.es (A.M.G.-C.); 2 Department Analytical Chemistry, Faculty of Chemistry, Regional Campus of International Excellence “Campus Mare Nostrum”, University of Murcia, E-30100 Murcia, Spain; 3 Department Animal Production, Faculty of Veterinary, Campus Univ. Rabanales, University of Córdoba, 14071 Córdoba, Spain; vrestevez@uco.es

**Keywords:** feed, pig, mycotoxins, liquid chromatography, fluorescence detection, mass spectrometry, solid-liquid extraction, co-occurrence

## Abstract

A survey including 228 pig feed samples from Spain has been developed, exploring the occurrence of 19 mycotoxins (aflatoxins B1, B2, G1 and G2, ochratoxin A, fumonisins B1 and B2, citrinin, zearalenone, deoxynivalenol, fusarenon X, sterigmatocystin, T-2 toxin, HT-2 toxin, enniatins A, A1, B and B2, and beauvericin). The samples were analysed by solid-liquid extraction followed by liquid chromatography coupled with fluorescence or mass spectrometry detection. Enniatin B was found in 100% of the samples (up to 1200 µg/kg) and beauvericin in more than 90%. Moreover, 40% of samples were contaminated with more than five mycotoxins. This high occurrence is insurmountable and surpasses all previous studies, probably due to the inclusion of emerging mycotoxins, scarcely explored. The majority of the samples (96.9%) were in accordance with EU regulations, which do not address emerging mycotoxins or co-occurrence. These results show that in order to ensure mycotoxin absence, emerging mycotoxins should always be considered.

## 1. Introduction

The European Union (EU) ensures the safety of foods by setting maximum levels of different contaminants including mycotoxins, which are toxic secondary metabolites produced by certain fungi (as *Aspergillus, Fusarium* or *Penicillium*) that can contaminate food during harvesting, processing or storage. Among them, there are well-known hazards such as aflatoxins (AFs), considered as carcinogenic to humans by the International Agency for Research on Cancer [1] or ochratoxin A (OTA) and fumonisin B1, considered as possibly carcinogenic to humans [2,3]. Currently, several mycotoxins are included in the EU legislation and maximum contents have been established in different raw materials and food commodities. However, despite the effort to control these food contaminants, every year mycotoxins are found among the “top ten” hazards reported annually by the Rapid Alert System for Food and Feed (RASFF), aflatoxins being the most frequently reported [4]. Moreover, there are still some mycotoxins without maximum content allowed in regulation, but with some evidence of adverse effects on human health. These are the so-called “emerging mycotoxins”, including some *Fusarium* toxins such as enniatins (ENNs) and beauvericin (BEA).

In addition to food, it is also important to control contamination in feeds, as they are the first link in the food-chain, having influence not only on the health of animals but also on humans consuming animal-derived products. To deal with feed safety, the EU has established regulatory levels for aflatoxin B1 (AFB1) and guidelines for deoxynivalenol (DON), zearalenone (ZEA), OTA, fumonisin B1 and B2 (FB1 and FB2), and T-2 and HT-2 toxins in different raw materials and feed, including pig diets (Table 1) [5,6,7]. Moreover, the European Food Safety Authority (EFSA) has published different scientific opinions about the risks to animal health related to the presence of different mycotoxins [8]. One of these opinions about the risks to human and animal health, related to the presence of BEA and ENNs in food and feed, concluded that in the absence of toxicological data for most livestock animals, research studies on their adverse effects are required [9]. 

Given their relatively high susceptibility to *Fusarium* toxins and the high content of cereals in their diet, pigs should be given the highest priority. Thus, several studies have shown the toxic effect of mycotoxins on pig health, including a modulation of the immune response, resulting in an increase in susceptibility and severity of infectious diseases, and a reduction in vaccine efficacy, whilst also having an indirect effect on animal productivity [10]. Especially abundant are the number of studies about the pathological effects of *Fusarium* toxins on pig reproduction [11]; these include abortion, embryonic and foetal death, increased number of oestrus repetitions, failure of induction programs with PGF2α and increased number of stillbirths and splaylegged piglets. What is more, ZEA can produce hyperestrogenism [12] and tail necrosis in suckling piglets [13]. The effects of DON on gut function [14,15], and DON and ZEA on colon microbiota, have also been reported [16]. Moreover, diets co-contaminated with AFs and FBs negatively affected the growth of piglets, despite the absence of pathology and despite the absence of clinical signs [17].

Different studies and reviews have reported the occurrence of mycotoxins in raw materials and feed, showing that multi contamination is very frequent and seems to be normal and not exceptional [18,19,20,21,22,23,24,25,26,27]. However, most of these studies are focused on regulated mycotoxins and only a few studies included emerging mycotoxins [28,29,30]. Nevertheless, EFSA has concluded that further data on the co-occurrence of different mycotoxins, including BEA and ENNs with other toxins in feed, and the probable combined effects are needed [9].

The aim of this work was to evaluate the occurrence of nineteen different mycotoxins, including *Fusarium* emerging mycotoxins, in pig feed from Spain. With this purpose, 228 samples were collected during 2017 from different farms and analysed using high performance liquid chromatography with post-column photochemical derivatisation and fluorescence detection (HPLC-FLD) for quantification of AFs and ultra-high performance liquid chromatography with tandem mass spectrometry (UHPLC-MS/MS) for the rest, while solid-liquid extraction was used as sample treatment. 

## 2. Results and Discussion

### Ocurrence of Mycotoxins in Pig Feed Samples

**Figure 1:**
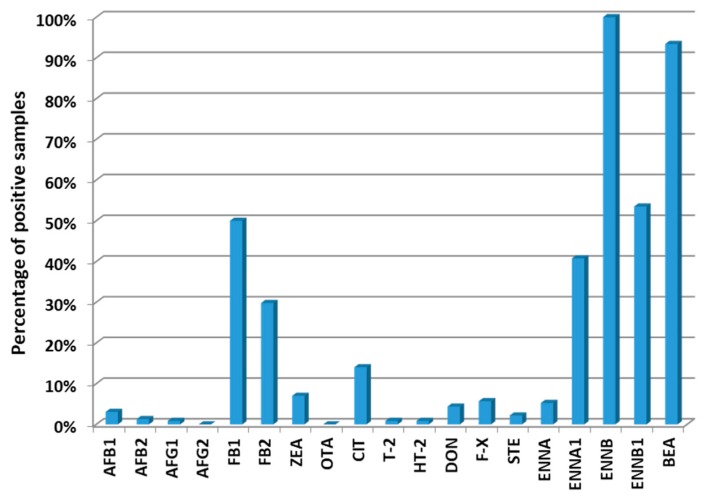
Percentage of positive samples of each mycotoxin.

Among the 19 mycotoxins under study, the most commonly found (considering samples with concentrations above the LOQs) were: ENNB (100% samples), BEA (93.4%), ENNB1 (53.5%), FB1 (50.0%) and ENNA1 (40.8%). Besides that, none of the samples presented concentrations above the LOD for AFG2 and OTA. Figure 1 summarises the results. This high mycotoxin occurrence surpasses all previous studies of occurrence; i.e., Streit et al. [18] found 72% positive samples for at least one mycotoxin when analysing 17,316 samples of feed and feed raw materials from all over the world to study contamination with AFs, OTA, ZEA, DON and fumonisins. However, other regional and national studies of occurrence have found higher occurrences for some mycotoxins above the LOD; i.e., Li et al. [31] detected DON and ZEA at percentages of 97 and 100% in samples of complete feeds randomly collected from 15 pig farms in the Beijing region (China), and Ma et al. [24] found AFB1, ZEA and DON at percentages from 96.4 to 100% also in samples of complete feeds from different provinces of China. The maximum incidence reached in the present study is probably due to the high number of mycotoxins analysed, including emerging mycotoxins, scarcely explored in other studies. This fact shows that for both a correct diagnosis and in order to ensure its absence, it is not enough to analyse only the most common mycotoxins.

Concerning the concentrations found, the contents of the two mycotoxins with higher incidence ranged from 2.0 to 1222 µg kg^−1^ for ENNB, and from 1.7 to 747 µg kg^−1^ for BEA in positive samples (100% and 93.4% respectively). However, the mycotoxin detected at the highest level was FB1 with a concentration of 3959 µg kg^−1^. A summary of all the results obtained is shown in Table 2. As can be seen from the high values of the %RSD, a great difference in the content of the different mycotoxins was observed among the samples.

The EU has established recommended guidance values on raw materials, complementary and complete feeding stuff with differences associated to age of animal and phase of production. Focusing the attention on those mycotoxins with maximum permitted or recommended levels, it can be concluded that all the samples fulfil the requirements about maximum content of AFB1, as well as the guidelines for OTA, FB1+FB2, T-2+HT-2 toxins, and DON. However, several samples (3.1%) showed contents of ZEA above the recommended levels: one sample of maize (7681 µg kg^−1^); five samples of compound feed for piglets (ranging from 125 to 956 µg kg^−1^), and one for fattening pigs (290 µg kg^−1^). In a recent study, it has been proven that growing pigs (six-week-old and 19 kg BW at the beginning) fed ad libitum with feed containing 80 µg kg^−1^ ZEA for four weeks reduced body weight gain, daily feed intake, feed conversion rate, and the serum levels of immunoglobulin IgG and IgM, and total antioxidants, showing microscopic lesions in kidneys [32]. This study shows the need to reduce the levels of tolerance for ZEA in feeds for pigs.

These results are in accordance with other survey studies carried out in Europe, where *Fusarium* toxins, such as DON, ZEA, and FBs, were the most frequently found mycotoxins in feed [23].

**Figure 2:**
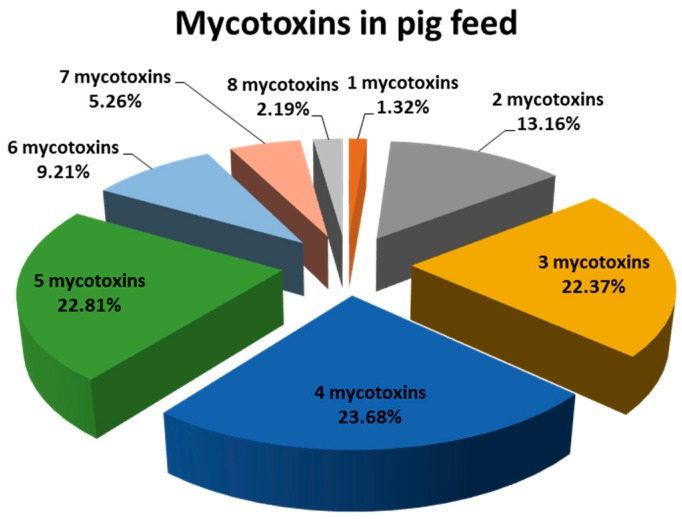
Frequency and co-occurrence of different mycotoxins in feed samples.

Another aspect of concern was the high co-occurrence of mycotoxins. Thus, 69% of analysed samples contained between 3 and 5 mycotoxins (Figure 2), the most common combinations being those involving co-occurrence of different emerging mycotoxins: ENNB + BEA (12.7%); ENNB + ENNB1 + BEA (8.3%) and ENNA1 + ENNB + ENNB1 + BEA (10.5%). Moreover, the co-occurrence of more than one regulated mycotoxin (AFs, OTA, FBs, DON, ZEA, and T-2 + HT-2) was detected in 19 samples (8.3% of all samples). The natural co-occurrence of mycotoxins in feeds is already known and several surveys have reported this everywhere [21]; this can be explained by these diets being usually made up of multiple cereal and other grain sources; and mycotoxin frequency and levels in feed depend on these raw materials and, especially, on their geographic origin [21,24,31]; i.e., a study of 17,316 samples of feed and feed raw materials from all over the world showed 38% samples co-contaminated [18]. Although several reviews of existing data and of the literature on worldwide mycotoxin contamination of food and feed are available, the impact of the different raw materials used on feed formulation has not been widely studied. Concerning cereals and derived cereal product samples, 127 mycotoxin combinations were described by Smith et al., showing that 70%, 24% and 6% of the studies concerning the effect of co-occurrence of mycotoxins were binary, ternary and quaternary or quinary mixtures, respectively [33]. However, this level of study is not proportional to the co-occurrence found in the present study with 13.16%, 22.37% and 46.49% for binary, ternary and quaternary or quinary mixtures, respectively. Nevertheless, the current regulations do not consider the mycotoxin combination effects and the maximum allowed or recommended levels are fixed for a single mycotoxin. These results should be a matter of concern, as additive or synergistic effect (still not well-known) can increase the toxicity of mycotoxins. Hence, guidelines should not only be individually set for each mycotoxin but also for especially concerning combinations, particularly for those including emerging mycotoxins such as moniliformin, BEA or ENNs [22]. This fact has also been pointed out in a recent review compiling in vitro experimental data on mycotoxins combined toxicity [33]. Besides, more stringent mycotoxin limits of tolerance should be established, especially when there is a risk of chronic ingestion of regular contaminants of pig feed [32].

In order to elucidate which factors were the most relevant, one-way ANOVA studies were carried out for total content of mycotoxins as well as for each mycotoxin individually, considering different factors.

- *PRESENTATION.* ANOVA showed that no significant differences for total mycotoxin content (flour: 467 ± 549 µg kg^−1^; pellet: 404 ± 360 µg kg^−1^; *p*-value: 0.4741) existed between both presentations, however higher concentration of sterigmatocystin (STE) was found in pellet (flour: 0.193 ± 1.50 µg kg^−1^; pellet: 11.3 ± 53.1; *p*-value: 0.0053).
- *SAMPLING POINT.* This factor was significant for total mycotoxin content (silo: 436 ± 607 µg kg^−1^, feeder: 244 ± 160 µg kg^−1^, sack: 677 ± 554 µg kg^−1^, bulk: 3091 ± 4075 µg kg^−1^, *p*-value: 0.0000). In addition, significant differences were obtained for citrinin (CIT) (silo: 11 ± 31 µg kg^−1^, hopper: 22 ± 50 µg kg^−1^, sack: 70 ± 135 µg kg^−1^, bulk: 0 ± 0 µg kg^−1^, *p*-value: 0.0022), DON (silo: 14 ± 56 µg kg^−1^, hopper: 0 ± 0 µg kg^−1^, sack: 0 ± 0 µg kg^−1^, bulk: 93 ± 207 µg kg^−1^, *p*-value: 0.0074), FB1 (silo: 150 ± 353 µg kg^−1^, hopper: 12 ± 38 µg kg^−1^, sack: 323 ± 399 µg kg^−1^, bulk: 1278 ± 1462 µg kg^−1^, *p*-value: 0.0000), FB2 (silo: 0 ± 0 µg kg^−1^, hopper: 45 ± 123 µg kg^−1^, sack: 71 ± 105 µg kg^−1^, bulk: 339 ± 362 µg kg^−1^, *p*-value: 0.0000) and ZEA (silo: 41 ± 137 µg kg^−1^, hopper: 0 ± 0 µg kg^−1^, sack: 32 ± 132 µg kg^−1^, bulk: 1297 ± 2855 µg kg^−1^, *p*-value: 0.0001). In all the cases, higher concentrations were obtained for samples from bulk storage without container in warehouse except for CIT, where the highest concentrations were found in sack. The higher mycotoxin content could be explained because bulk storage without container is less hygienic and the feed is in contact with the warehouse floor. Sampling is usually the principal source of variation in toxin analysis, originating close to 90% of the error in some analyses [34]. Traceability is important and sampling of feed should cover from factory to feeder, including silos and warehouses.
- *TYPE OF ANIMAL.* These feeds have different proportions of raw materials to provide different nutrient levels; however, cereal grains are always the main component. ANOVA results in no significant differences among the feeds for the total mycotoxin content and for any isolated mycotoxin; the high proportion of cereals in all these feeds could explain the lack of differences. However, less contamination could be expected in piglet feed than in older pig feed, which could be explained by the lower level of maize used for piglets diet, maize being the main contributor to mycotoxin contamination [21].

## 3. Conclusions

A survey of nineteen mycotoxins (including emerging mycotoxins) in pig feed samples collected during 2017 from different farms and suppliers from Spain has been carried out. The methods of analysis were fully validated, using HPLC-FLD for AFs and UHPLC-MS/MS for the remaining mycotoxins as analytical techniques, while solid-liquid extraction was used as sample treatment. All the samples fulfilled the requirement about the maximum or recommended content of AFB1, OTA, FB1+FB2, T-2+HT-2 and DON. However, several samples showed contents of ZEA above the recommended levels.

This survey on mycotoxin occurrence in pig feed contains one of the most complete analyses, quantifying 19 different mycotoxins, including five emerging ones. Although compliance with EU regulations has been generally high, the study showed the high occurrence of emerging mycotoxins (100% contaminated samples), as well as the high co-occurrence of different mycotoxins in the same sample.

Livestock species respond differently to mycotoxin poisoning, so that clinical signs can be difficult to detect and a correct analysis of the food is the most appropriate way to diagnose and detect it. Moreover, the synergistic interaction of co-occurring mycotoxins is well-known, hence regulations for maximum levels should be set including some particular combinations, focusing on emerging mycotoxins such as ENNs. These results highlight the necessity for a reinforcement of quality control of feeds with continuous monitoring from feed mill to pig feeder.

## 4. Materials and Methods

### 4.1. Chemicals and Reagents

All reagents were of analytical reagent grade and solvents were HPLC grade. Acetonitrile (MeCN), methanol (MeOH) and ammonium formate were obtained from VWR BDH Prolabo (West Chester, PA, USA). Formic acid was supplied by Merck (Darmstadt, Germany). Sodium chloride, magnesium sulfate, sodium citrate, disodium hydrogen citrate sesquihydrate and sodium phosphate dihydrogen monohydrate were purchased from Panreac Química (Barcelona, Spain).

Ultrapure water used throughout the work was obtained from a Milli-Q water purification system (18.2 MΩ cm-1, Milli-Q Plus system, Millipore, Bedford, MA, USA).

AFs (AFB1, AFB2, AFG1, AFG2) were purchased from Sigma-Aldrich (St. Louis, MO, USA); OTA, DON, ZEA, CIT, T-2, HT-2, fusarenon-X (F-X), STE, FB1, FB2 and ENNs (ENNA, ENNB and ENNB1) were purchased from Techno Spec (Barcelona, Spain), while BEA and ENNA1 were supplied by VWR International Eurolab, S.L. (Barcelona, Spain). Individual stock standard solutions were prepared for all of them in MeCN and kept in the dark at −18 °C. 

Different intermediate solutions were prepared due to the differences in the analytical method and sample treatment. The first solution was composed of ENNA, ENNA1, ENNB, ENNB1 and BEA at a concentration of 10 μg mL^−1^. The second solution was composed of FB1 and FB2, DON, ZEA, CIT, OTA, T-2, HT-2, F-X and STE at a concentration of 1 μg mL^−1^ each mycotoxin. The third solution was composed of AFB1, AFB2, AFG1 and AFG2 at a concentration of 50 μg mL^−1^. Working solutions were prepared by diluting the intermediate solutions in MeOH:water (50:50).

Syringe filters (25 mm, 0.2 µm nylon membrane, VWR, West Chester, PA, USA) were used for filtration of samples prior to the injection into the chromatographic system.

### 4.2. Instrumentation and Equipment

The determination of AFs was carried out using a modular HPLC system consisting of a quaternary low pressure gradient pump (Model PU-2089, Jasco, Tokyo, Japan); an autosampler with a 100 µL loop (Model AS-2055, Jasco, Tokyo, Japan); a C18 Kinetex separation column (150 mm × 4.6 mm, 2.6 µm) from Phenomenex (Torrance, CA, USA) placed in a column oven; a UV derivatization module, which consists of a photochemical reactor specific for the analysis of aflatoxins with a 254 nm lamp (LCTech, Dorfen, Germany) and finally, a fluorescence detector (Model FP 2020, Jasco, Tokyo, Japan) used to acquire the signals. ChromNAV software (1.09.03 version, Jasco, Tokyo, Japan) was used for data acquisition and processing of AFs.

In order to determine the remaining mycotoxins an Agilent 1290 Infinity LC system (Agilent Technologies, Waldbronn, Germany) equipped with a binary pump, an on-line degasser, autosampler and a column oven placing a Zorbax Eclipse Plus RRHD C18 column (50 mm × 2.1 mm, 1.8 µm) coupled to a triple quadrupole (QqQ) mass spectrometer API 3200 (AB SCIEX, Toronto, ON, Canada) with electrospray ionization (ESI) was used. Data were processed using the Analyst Software version 1.5 with schedule multiple reaction monitoring (MRM) TM Algorithm (AB SCIEX).

For sample treatment, an evaporator system (System EVA-EC, from VLM GmbH, Bielefeld, Germany), a vortex-2 Genie (Scientific Industries, Bohemia, NY, USA) and a universal 320R centrifuge (HettichZentrifugen, Tuttlingen, Germany) were used.

### 4.3. Samples

A total of 228 samples of pig feed were collected from February to August 2017 at different farms and manufacturing industries around Spain. Samples (approximately 500 g) were sent directly to the laboratory by the veterinarians responsible for farms or feed mills. Analytical personnel were not present to oversee the sampling. They included: 71 compound feed samples destined to fattening pigs, 42 to sows, 111 to piglets, 2 to gilts, and 2 samples of maize (grain given to the fattening pigs in two farms). The information of each sample comprised: animals to which it was destined, presentation (flour or pellet) and sampling point (silo, feeder, sack and bulk storage without container in warehouse). Samples were milled and homogenised using a standard grinder. Finally, all samples were stored at room temperature until analysis.

### 4.4. Sample Preparation

Two different extractions were carried out: one for determination of AFs by HPLC-FLD and the other for the rest of mycotoxins by UHPLC-MS/MS.

#### 4.4.1. Aflatoxins

A previously reported method was adapted [35]. Briefly, 2 g of homogenised sample were placed into a 50-mL screw cap test tube with conical bottom. The sample was extracted with 10 mL of MeCN, shaking by vortex for 3 min. The sample was centrifuged at 4500 rpm for 5 min. Afterwards, 2 mL of the upper layer was pipetted into a vial and dried under a gentle stream of N2. Finally, the residue was dissolved with 1 mL of MeOH:water (50:50, *v*/*v*) and the solution thus obtained was filtered through a 0.22 µm nylon membrane filter before injection into the HPLC-FLD system.

#### 4.4.2. Multimycotoxins, Enniatins and Beauvericin

Two g of homogenised sample and 8 mL of water were placed into a 50-mL screw cap test tube with conical bottom, shaken by vortex for 1 min. Then, 10 mL of solvent (MeCN, 5% formic acid) were added to the tube, shaken by vortex for 3 min, following by the addition of salts (4 g MgSO_4_, 1 g NaCl), shaken vigorously for 2 min. After these steps, the tubes were centrifuged at 4500 rpm for 5 min. Finally, 2 mL of the organic layer was evaporated to dryness under a gentle stream of N_2_. The residue was dissolved with 1 mL of MeOH:water (50:50, *v*/*v*). Each solution was filtrated with 0.22 µm membrane filter prior to the UHPLC-MS/MS analysis.

### 4.5. Chromatographic Separation and Detection

In order to achieve the lowest limits of quantification, determination of the different mycotoxins was carried out in three different analyses: AFs were determined by HPLC-FLD, multimycotoxins by UHLC-MS/MS and emerging mycotoxins (ENNs and BEA) in a different UHPLC-MS/MS analysis.

#### 4.5.1. Determination of Aflatoxins by HPLC-FLD

The chromatographic separation was carried out according to Arroyo-Manzanares et al. [35]. The chromatographic separation was achieved using a C18 Kinetex separation column (150 mm × 4.6 mm, 2.6 µm). The mobile phase consisted of three different solvents: eluent A (water), eluent B (MeOH) and eluent C (MeCN) and a linear gradient elution was used as follows, keeping constant eluent B at 27%: 0–3 min: 0% C; 20 min: 13% C; and 21–23 min: 68% C, followed by an equilibration time of 10 min. The injection volume was 50 µL and the flow rate was 1 mL min^−1^. The temperature of the column was kept constant at 30 °C. Excitation and emission wavelengths of the FLD for the determination of the aflatoxins were set at 365 nm and 460 nm, respectively. The FLD was working at gain x100.

#### 4.5.2. Determination of Multimycotoxins by LC-MS/MS

Conditions similar to those reported in a previous paper were used [36]. The chromatographic separation of multimycotoxins (OTA, FB1, FB2, T-2, HT-2, STE, CIT, F-X, DON, ZEA) was carried out on a C18 Zorbax Eclipse Plus RRHD column (50 × 2.1 mm, 1.8 µm) and as mobile phase eluent A (5 mM ammonium formate aqueous solution, 0.3% formic acid), and eluent B (MeOH with 5 mM ammonium formate, 0.3% formic acid), under the following gradient conditions: 0 min: 5% B; 1 min: 50% B; 4 min: 80% B; 6 min: 90% B, 6.20 min: 5% B and 8 min: 5% B. The injection volume was 5 µL, the flow rate was 0.4 mL min^−1^ and the temperature of the column was 35 °C.

MRM conditions of the mass spectrometer for the determination of multimycotoxins are shown in Supplementary Materials (Table S1). The working conditions of the mass spectrometer were established in positive ESI mode under the following conditions: temperature of the source 500 °C; voltage of the ion spray 5 KV, collision gas (nitrogen) 5 psi; curtain gas (nitrogen) 30 psi; GAS 1 and GAS 2 (both of them nitrogen) 50 psi. In all circumstances, an ion was used as precursor and two ions were obtained from the fragmentation process: the most abundant one for quantification (Q) and the other for confirmation (I).

#### 4.5.3. Determination of Enniatins and Beauvericin by LC-MS/MS

The same column (C18 Zorbax Eclipse Plus RRHD) was used for separation of the five emerging mycotoxins. However, the mobile phase did not include ammonium formate in its composition in order to avoid the formation of ammonium adducts. Thus, this mobile phase consisted of eluent A (0.3% formic acid aqueous solution), and eluent B (MeOH with 0.3% formic acid). The following linear gradient elution was used: 0 min: 70% B; 2 min: 90% B; 4 min: 90% B; 4.2 min: 70% B, and 6 min: 70% B. The flow rate was 0.4 mL min^−1^. The injection volume was 5 µL and the column temperature was 35 °C.

MRM conditions of the mass spectrometer are shown in the Supplementary Materials (Table S1). The working conditions of the mass spectrometer were established in positive ESI mode, working under the conditions described for multimycotoxin determination.

### 4.6. Method Development

#### Optimisation of Sample Preparation

The extraction of AFs was performed according to a previously reported method [35], so no further optimisation was required.

Extraction of multimycotoxins and emerging mycotoxins was optimised, trying to obtain one single sample treatment suitable for the extraction of all mycotoxins. With this purpose, solid-liquid extraction with and without partitioning process were tested using 2 g of sample, as follows: (a) 10 mL MeCN with 5% formic acid; (b) 10 mL MeOH with 5% formic acid; (c) 8 mL H_2_O + 10 mL MeCN with 5% formic acid + 4 g MgSO_4_ + 1 g NaCl; (d) 8 mL H_2_O + 10 mL MeCN with 5% formic acid + 4 g MgSO_4_ + 1 g NaCl + 1 g sodium citrate + 0.5 g disodium hydrogen citrate sesquihydrate; (e) 8 mL 30 mM NaH_2_PO_4_ buffer pH 7.1 +10 mL MeCN with 5% formic acid + 4 g MgSO_4_ + 1 g NaCl + 1 g sodium citrate + 0.5 g disodium hydrogen citrate sesquihydrate; and (f) 8 mL H_2_O + 10 mL MeCN with 5% formic acid + 4 g MgSO_4_, 1 g NaCl). The best results in terms of recovery were obtained with conditions described in (c).

Subsequently, the volume of extraction solvent (MeCN, 5% formic acid) was studied within 5 and 10 mL, 10 mL being chosen as optimum in view of the worst recoveries if lower extraction volumes were utilised. Afterwards, the volume of extract to dry was studied, choosing a volume of 2 mL as a compromise to achieve a short time of sample treatment and low limits of detection (LODs).

### 4.7. Method Validation

The validation of each method comprised the establishment of the calibration curves in the presence of matrix, evaluation of LODs and limits of quantification (LOQs), selectivity, precision (as interday and intraday precision at different concentration levels) and extraction recovery studies at different concentration levels. In all the cases, a sample of feed previously analysed and free of mycotoxins was chosen as the representative matrix.

#### 4.7.1. Aflatoxins

As stated previously, this method had been previously validated for different kinds of feeds, including pig feeding [35]. The method provided good linearity in the concentration range 1–50 µg kg^−1^ (*R^2^* > 0.99), LOQs of 1 µg kg^-1^, precisions expressed as RSD lower than 7.9% and 9.2% for intraday and interday studies, respectively, and recoveries higher than 85%.

#### 4.7.2. Multimycotoxins

For the evaluation of linearity, calibration curves were obtained by spiking portions of a representative feed sample with different concentrations of analytes, depending on the analytical sensitivity for each mycotoxin. Samples were submitted to the sample treatment and analysed and the area of the Q ion was selected as analytical signal. LODs and LOQs were calculated as 3 × S/N and 10 × S/N, respectively. The results are shown in Table 3.

The matrix effect was evaluated by relative comparison of the analyte peak area of a spiked extract of sample and a standard solution at the same concentration. Ion suppression was observed for most of the analytes, so calibration in presence of matrix was mandatory for quantification. These studies, as well as intra and interday precision and recovery studies, were carried out at three different concentration levels (three samples, injected in triplicate). The results are shown in Supplementary Materials (Tables S2 and S3).

#### 4.7.3. Enniatins and Beauvericin

A similar procedure was followed for validation of UHPLC-MS/MS determination of emerging mycotoxins. Calibration curves were obtained by spiking a feed sample with concentrations of analytes up to 500 µg kg^−1^, submitted to the whole procedure and analysed. Table 3 summarises the results. 

The matrix effect, precision and trueness were evaluated at three different concentration levels (three samples, injected in triplicate). Once again, ion suppression was observed for all the analytes, so calibration in presence of matrix was mandatory for quantification. The results are shown in Supplementary Materials (Tables S2 and S3).

### 4.8. Data Processing

One-way ANOVA studies were carried out for total content of mycotoxins as well as for each mycotoxin individually, considering different factors, namely: presentation (pellet or flour), sampling point (silo, feeder, sack and bulk storage without container in warehouse), and type of animal feed (piglet, gilts, sows and fattening pigs). When this information was not available, the sample was not included in the study. These studies were performed to determine statistical differences with a level of confidence of 95%. 

*PRESENTATION.* A total of 226 samples were analysed, 183 presented as flour and 43 as pellet (*n* = 43). The two samples of maize (grain) were excluded from this study.

*SAMPLING POINT.* In this case, a total of 137 were processed, 77 samples were collected from a silo, 29 from a feeder, 25 from a sack and 6 were stored in bulk without container in warehouse. 

*TYPE OF ANIMAL.* Another factor is the type of pig for which the compound feed is intended. In this sense, different types of feed (classified according to the necessities of the animal) can be distinguished: piglets (*n* = 111), fattening (*n* = 71), gilts (*n* = 2) and sows (*n* = 42). The two samples of maize (grain) were excluded from this study.

## Figures and Tables

**Table 1:** Maximum content (AFB1) or recommended levels for mycotoxins in feed (only those concerning swine) [5,6,7].

Mycotoxin	Product Intended for Pig Feed	Maximum/Guidance Value (µg kg^−1^)
**AFB1**	Feed materials	20
	Complementary and complete feed	10
	Compound feed for pigs (except piglets)	20
	Compound feed for piglets	5
**DON**	Maize by-products	12,000
	Cereals and cereal products with the exception of maize by-products	8000
	Maize by-products	12,000
	Complementary and complete feedingstuffs for pigs	900
**ZEA**	Cereals and cereal products with the exception of maize by-products	2000
	Maize by-products	3000
	Complementary and complete feedingstuffs for piglets and gilts (young sows)	100
	Complementary and complete feedingstuffs for sows and fattening pigs	250
**OTA**	Cereals and cereal products	250
	Complementary and complete feedingstuffs for pigs	50
**FB1+FB2**	Maize and maize products	60,000
	Complementary and complete feedingstuffs for pigs	5000
**T-2+HT-2**	Oat milling products (husks)	2000
	Other cereal products	500
	Compound feed	250

**Table 2:** Summary of the occurrence of the studied mycotoxins: number and percentage of positive samples, mean concentration value of positive samples, median, 1st and 3rd quartile of positives samples, RSD% and minimum and maximum found concentrations.

	Nº Positive Samples	Incidence (%)	Mean (µg kg^−1^)	%RSD	Min (µg kg^−1^)	Max (µg kg^−1^)	0.25th Quantile (µg kg^−1^)	0.50th Quantile (µg kg^−1^)	0.75th Quantile (µg kg^−1^)	Nº Non-Compliant Samples ^a^
**AFB1**	7	3.07	0.94	90	0.29	2.91	0.44	0.57	1.17	0
**A** **FB2**	3	1.32	0.60	55	0.28	1.06	0.28	0.47	1.06	---
**AFG1**	2	0.88	0.33	33	0.22	0.44	---	0.33	---	---
**AFG2**	0	0	--	--	--	--	---	---	---	---
**FB1**	114	50.00	403	133	4.09	3959	99.1	209	541	0
**FB2**	68	29.82	184	101	3.63	961	61.9	133	221	
**ZEA**	16	7.02	741	244	101	7681	126	137.0	463	7
**OTA**	0	0	--	--	--	--	---	---	---	0
**CIT**	32	14.04	147	80	10.7	512	71.0	109.0	197	---
**T-2**	2	0.88	31.9	12	28.0	35.9	---	31.9	---	0
**HT-2**	2	0.88	117	4.8	112	123	---	117	---	
**DON**	10	4.39	237	51	153	555	164	182	263	0
**F-X**	13	5.70	291	82	65.1	821	94.1	212	428	---
**STE**	5	2.19	104	115	11.2	308	11.3	12.8	243	---
**ENNA**	12	5.26	9.82	170	3.29	64.9	3.52	3.9	8.07	---
**ENNA1**	93	40.79	19.0	128	4.54	140	6.65	10.5	20.9	---
**ENNB**	228	100	118	137	2.01	1222	14.9	54.3	165	---
**ENNB1**	122	53.51	34.3	141	2.58	247	6.53	15.1	38.2	---
**BEA**	213	93.42	20.7	270	1.71	747	4.93	8.73	19.2	---

^a^ According to EU directives and recommendations [5,6,7].

**Table 3:** Calibration curves, LODs and LOQs for multimycotoxin, enniatins and beauvericin determination.

	Calibration Curve	Linear Range (µg kg^−1^)	*R* ^2^	LOD (µg kg^−1^)	LOQ (µg kg^−1^)
**DON**	y = 3.5279x + 100.67	86.1–1000	0.998	26	86
**F-X**	y = 1.0892x − 8.111	135.9–2000	0.995	41	136
**CIT**	y = 395.99x − 13280	24.3–500	0.999	7.3	24
**HT-2**	y = 8.555x − 29.894	33.9–2000	0.998	10	34
**FB1**	y = 11.103x + 347.53	22.2–500	0.999	6.7	22
**T-2**	y = 40.038x + 1245.4	26.8–2000	0.995	8.1	27
**ZEA**	y = 29.141x − 758.98	99.0–500	0.998	30	99
**OTA**	y = 82.66x + 333.23	8.1–100	0.993	2.5	8.1
**STE**	y = 208.73x − 848.16	9.9–100	0.991	3.0	9.9
**FB2**	y = 13.57x + 112.34	38.5–500	0.999	12	38
**ENNB**	y = 987.45x − 2943.8	2.00–500	0.998	0.60	2.0
**ENNB1**	y = 1482.5x + 9430.2	2.53–500	0.999	0.76	2.5
**BEA**	y = 372.32x + 0.0047	1.72–500	0.998	0.52	1.7
**ENNA1**	y = 1092.9x + 3989.6	4.52–500	0.999	1.4	4.5
**ENNA**	y = 1578.8x + 3790.5	2.94–500	0.999	0.88	2.9

## Supplementary Materials

The following are available online at https://www.mdpi.com/2072-6651/11/6/342/s1, Table S1: Monitored ions of multimycotoxins, enniatins and beauvericin, and MS/MS parameters; Table S2: Matrix effect (%ME), recovery (%R) and recovery precision (%RSD) for multimycotoxin enniatins and beauvericin determination (*n* = 9); Table S3: Precision study for multimycotoxin, enniatins and beauvericin determination at different concentration levels (*n* = 9).
