# Supplementary material for: Occurrence of Mycotoxins in Swine Feeding from Spain
Source: Toxins (Basel). 2019 Jun 15;11(6):342. doi: 10.3390/toxins11060342 (PMC6628415; doi:10.3390/toxins11060342)
Supplement: Supplementary file 1 [file toxins-11-00342-s001.pdf]

Supplementary Materials: Occurrence of Mycotoxins in Swine Feeding from Spain

Natalia Arroyo-Manzanares, Vicente Rodríguez-Estévez, Plácido Arenas-Fernández, Ana M. García-Campaña and Laura Gámiz-Gracia

Table S1. Monitored ions of multimycotoxins, enniatins and beauvericin, and MS/MS parameters.

Mycotoxin	Retention Time (min)	Precursor Ion (*m*/*z*)	Molecular Ion	DP [a]	EP [a]	CEP [a]	Product Ions [b]	CE [a]	CXP [a]
**DON**	1.70	297.1	$[M+H]^+$	36.0	5.5	16	249.2(Q)	17.0	4.0
							161.0 (I)	29.0	4.0
**F-X**	2.02	355.1	$[M+H]^+$	26.0	12.0	18	174.7(Q)	23.0	4.0
							137.1 (I)	31.0	4.0
**CIT**	2.90	251.2	$[M+H]^+$	26.0	11.0	18	233.0 (Q)	23.0	23.0
							204.8 (I)	73.0	10.0
**HT-2**	3.18	442.0	$[M+NH_4]^+$	21.0	5.5	21	262.8 (Q)	22.0	8.0
							215.4 (I)	19.0	4.0
**FB1**	3.24	722.2	$[M+H]^+$	71.0	10.0	30	334.2 (Q)	51.0	6.0
							352.2 (I)	47.0	6.0
**T-2**	3.44	484.0	$[M+NH_4]^+$	21.0	10.0	22	215.0 (Q)	22.0	4.0
							185.0 (I)	29.0	4.0
**ZEN**	3.71	319.0	$[M+H]^+$	26.0	8.0	20	282.9(Q)	15.0	10.0
							301.0 (I)	19.0	4.0
**OTA**	3.76	404.0	$[M+H]^+$	41.0	7.5	16	238.9 (Q)	31.0	6.0
							102.1 (I)	91.0	6.0
**FB2**	3.86	706.2	$[M+H]^+$	71.0	10.5	20	336.3 (Q)	43.0	14.0
							318.3 (I)	45.0	12.0
**STE**	3.88	325.1	$[M+H]^+$	66.0	3.5	26	281.0 (Q)	43.0	23.0
							310.0 (I)	37.0	4.0
**ENNB**	2.28	640.4	$[M+H]^+$	81.0	8.5	18.0	196.2 (Q)	35.0	4.0
							214.2 (I)	37.0	4.0
**ENNB1**	2.44	654.4	$[M+H]^+$	81.0	7.5	30.0	196.4 (Q)	39.0	6.0
							210.1 (I)	33.0	6.0
**BEA**	2.45	784.5	$[M+H]^+$	81.0	9.0	26.0	244.2 (Q)	39.0	4.0
							262.2 (I)	37.0	4.0
**ENNA1**	2.58	668.4	$[M+H]^+$	81.0	10.5	18.0	210.2 (Q)	35.0	6.0
							228.2 (I)	35.0	4.0
**ENNA**	2.74	682.4	$[M+H]^+$	76.0	9.0	26.0	210.2 (Q)	35.0	4.0
							228.2 (I)	35.0	4.0

[a] Declustering potential (DP), Entrance potential (EP), Collision Cell Entrance Potential (CEP), Collision Cell Exit Potential (CXP) and Collision Energy (CE). All expressed in voltage. [b] Product ions: (Q) transition used for quantification, (I) Transition employed to confirm the identification.

Table S2. Matrix effect (%ME), recovery (%R) and recovery precision (%RSD) for multmycotoxin enniatins and beauvericin determination (*n* = 9).

Mycotoxin	Level 1			Level 2			Level 3		
	%ME	%R	%RSD	%ME	%R	%RSD	%ME	%R	%RSD
DON	−36.7	51.6	2.1	−30.8	59.2	1.4	−43.3	55.2	6.9
F-X	−21.5	86.8	4.2	−28.8	90.8	5.5	−25.6	70.5	5.0
CIT	−66.7	74.4	4.7	−62.8	71.4	6.1	−63.3	73.2	5.9
HT-2	0.52	76.3	7.1	−0.30	79.5	6.0	−0.14	86.5	7.3
FB1	10.8	78.3	4.8	54.1	72.1	1.1	7.51	76.1	5.4
T-2	−27.8	82.9	5.5	−13.9	83.2	6.1	−9.51	77.5	5.9
ZEA	−37.7	84.2	6.1	−35.1	85.2	4.8	−44.4	92.1	5.9
OTA	−57.2	97.2	7.2	−60.8	95.1	6.7	−57.4	68.0	8.7
STE	−80.3	80.4	4.4	−77.1	86.5	7.0	−85.6	79.3	7.1
FB2	−9.3	73.6	4.2	7.84	77.1	3.6	−7.70	73.5	1.5
ENNB	−31.7	93.3	1.4	−70.4	99.7	0.9	−62.9	98.1	0.4
ENNB1	−73.6	93.4	1.1	−76.2	97.6	1.5	−71.5	100.2	1.3
BEA	−93.8	95.6	3.8	−94.6	99.0	1.0	−93.0	100.3	0.3
ENNA1	−82.5	97.5	1.4	−83.5	99.1	1.0	−80.2	99.6	0.2
ENNA	−89.0	98.2	0.3	−88.9	97.3	0.4	−86.6	99.7	2.3

Level 1 = OTA, STE: 10 μg kg^{-1}; HT-2, T-2, FB1, FB2, CIT: 50 μg kg^{-1}; ZEA: 100 μg kg^{-1}; F-X: 200 DON: 250 μg kg^{-1}; ENNA, ENNA1, ENNB, ENNB1, BEA: 20 μg kg^{-1}. Level 2 = OTA, STE: 50 μg kg^{-1}; HT-2, T-2, FB1, FB2, ZEA, CIT: 250 μg kg^{-1}; F-X, DON: 500 μg kg^{-1}; ENNA, ENNA1, ENNB, ENNB1, BEA: 100 μg kg^{-1}. Level 3 = OTA, STE: 100 μg kg^{-1}; HT-2, T-2, FB1, FB2, ZEA, CIT: 500 μg kg^{-1}; F-X: 1000 DON: 750 μg kg^{-1}; ENNA, ENNA1, ENNB, ENNB1, BEA: 200 μg kg^{-1}.

Table S3. Precision study for multimycotoxin, enniatins and beauvericin determination at different concentration levels (n = 9).

Mycotoxin	Intraday Precision, RSD%			Interday Precision, RSD%		
	Level 1	Level 2	Level 3	Level 1	Level 2	Level 3
**DON**	2.1	1.4	6.9	8.2	7.6	8.9
**F-X**	4.2	5.5	5.0	10	10	4.6
**CIT**	4.7	6.0	5.9	6.8	7.4	8.0
**HT-2**	7.1	6.0	7.3	4.2	9.8	8.5
**FB1**	4.8	1.1	5.4	4.3	3.5	7.8
**T-2**	5.5	6.1	5.9	2.1	2.2	3.1
**ZEA**	6.1	4.8	5.9	9.8	7.1	8.3
**OTA**	7.2	6.7	8.7	6.4	1.2	3.3
**STE**	4.4	7.0	7.1	3.4	7.5	6.3
**FB2**	4.2	3.6	1.5	5.4	5.9	7.3
**ENNB**	1.4	0.92	0.45	4.0	3.5	4.0
**ENNB1**	1.1	1.5	1.3	4.6	4.8	5.0
**BEA**	3.8	1.0	0.34	5.8	4.3	3.8
**ENNA1**	1.4	1.1	2.3	5.8	4.3	4.9
**ENNA**	0.29	0.38	0.18	4.8	3.3	4.9

Level 1 = OTA, STE: 10 μg kg^{-1}; HT-2, T-2, FB1, FB2, CIT: 50 μg kg^{-1}; ZEA: 100 μg kg^{-1}; F-X: 200 DON: 250 μg kg^{-1}; ENNA, ENNA1, ENNB, ENNB1, BEA: 20 μg kg^{-1}. Level 2 = OTA, STE: 50 μg kg^{-1}; HT-2, T-2, FB1, FB2, ZEA, CIT: 250 μg kg^{-1}; F-X, DON: 500 μg kg^{-1}; ENNA, ENNA1, ENNB, ENNB1, BEA: 100 μg kg^{-1}. Level 3 = OTA, STE: 100 μg kg^{-1}; HT-2, T-2, FB1, FB2, ZEA, CIT: 500 μg kg^{-1}; F-X: 1000 DON: 750 μg kg^{-1}; ENNA, ENNA1, ENNB, ENNB1, BEA: 200 μg kg^{-1}.
